# Value of addition of coronary artery calcium to risk scores in the prediction of major cardiovascular events in patients with type 2 diabetes

**DOI:** 10.1186/s12872-021-02352-4

**Published:** 2021-11-13

**Authors:** Barak Zafrir, Walid Saliba, Rachel Shay Li Widder, Razi Khoury, Elad Shemesh, David A. Halon

**Affiliations:** 1 Department of Cardiology, Lady Davis Carmel Medical Center, 7 Michal St., Haifa, Israel; 2 Community Medicine and Epidemiology, Lady Davis Carmel Medical Center, Haifa, Israel; 3 Cardiovascular Clinical Research Institute, Lady Davis Carmel Medical Center, Haifa, Israel; 4 Faculty of Medicine, Technion, Israel Institute of Technology, Haifa, Israel

**Keywords:** Diabetes mellitus, Coronary artery calcium, Risk stratification, Cardiovascular disease

## Abstract

**Background:**

The increased risk for cardiovascular events in diabetics is heterogeneous and contemporary clinical risk score calculators have limited predictive value. We therefore examined the additional value of coronary artery calcium score (CACS) in outcome prediction in type 2 diabetics without clinical coronary artery disease (CAD).

**Methods:**

The study examined a population-based cohort of type 2 diabetics (n = 735) aged 55–74 years, recruited between 2006 and 2008. Patients had at least one additional risk factor and no history or symptoms of CAD. Risk assessment tools included Pooled Cohort Equations (PCE) and Multi-Ethnic Study of Atherosclerosis (MESA) 10-year risk score calculators and CACS. The occurrence of myocardial infarction (MI), stroke or cardiovascular death (MACE) was assessed over 10-years.

**Results:**

Risk score calculators predicted MACE and MI and cardiovascular death individually but not stroke. Increasing levels of CACS predicted MACE and its components independently of clinical risk scores, glycated hemoglobin and other baseline variables: hazard ratio (95% confidence interval) 2.92 (1.06–7.86), 6.53 (2.47–17.29) and 8.3 (3.28–21) for CACS of 1–100, 101–300 and > 300 Agatston units respectively, compared to CACS = 0. Addition of CACS to PCE improved discrimination of MACE [AUC of PCE 0.615 (0.555–0.676) versus PCE + CACS 0.696 (0.642–0.749); *p* = 0.0024]. Coronary artery calcium was absent in 24% of the study population and was associated with very low event rates even in those with high estimated risk scores.

**Conclusions:**

CACS in asymptomatic type 2 diabetics provides additional prognostic information beyond that obtained from clinical risk scores alone leading to better discrimination between risk categories.

**Supplementary Information:**

The online version contains supplementary material available at 10.1186/s12872-021-02352-4.

## Background

Diabetes mellitus is associated with more complex and progressive coronary atherosclerosis, leading to the development of cardiovascular disease (CVD) [1, 2]. However, cardiovascular risk in diabetics is heterogeneous [3] and clinical risk scores are limited in defining individual risk and thus intensity of preventive measures required. Non-invasive imaging modalities, particularly non-contrast enhanced computed tomography (CT) coronary artery calcium scoring (CACS), may improve the ability to risk stratify diabetics [4]. Past studies examined the additive prognostic value of CACS in diabetics in comparison to older risk score models [5, 6]. In recent years, the Pooled Cohort Equation (PCE) and the Multi-Ethnic Study of Atherosclerosis (MESA) risk scores, which both include diabetes, are more widely used in clinical practice for assessing future risk of cardiovascular events [7, 8]. However, limited data exist in diabetics regarding the additional predictive value of combining these risk models with CACS. Moreover, although it is widely recognized that CACS can improve clinical risk assessment in asymptomatic individuals, its routine use in diabetics is debated [9]. In the current study, we aimed to investigate the performance of the PCE and MESA risk scores with or without CACS in predicting long-term cardiovascular outcomes of diabetic patients without coronary artery disease (CAD).

## Methods

### Study population

**Fig. 1:**
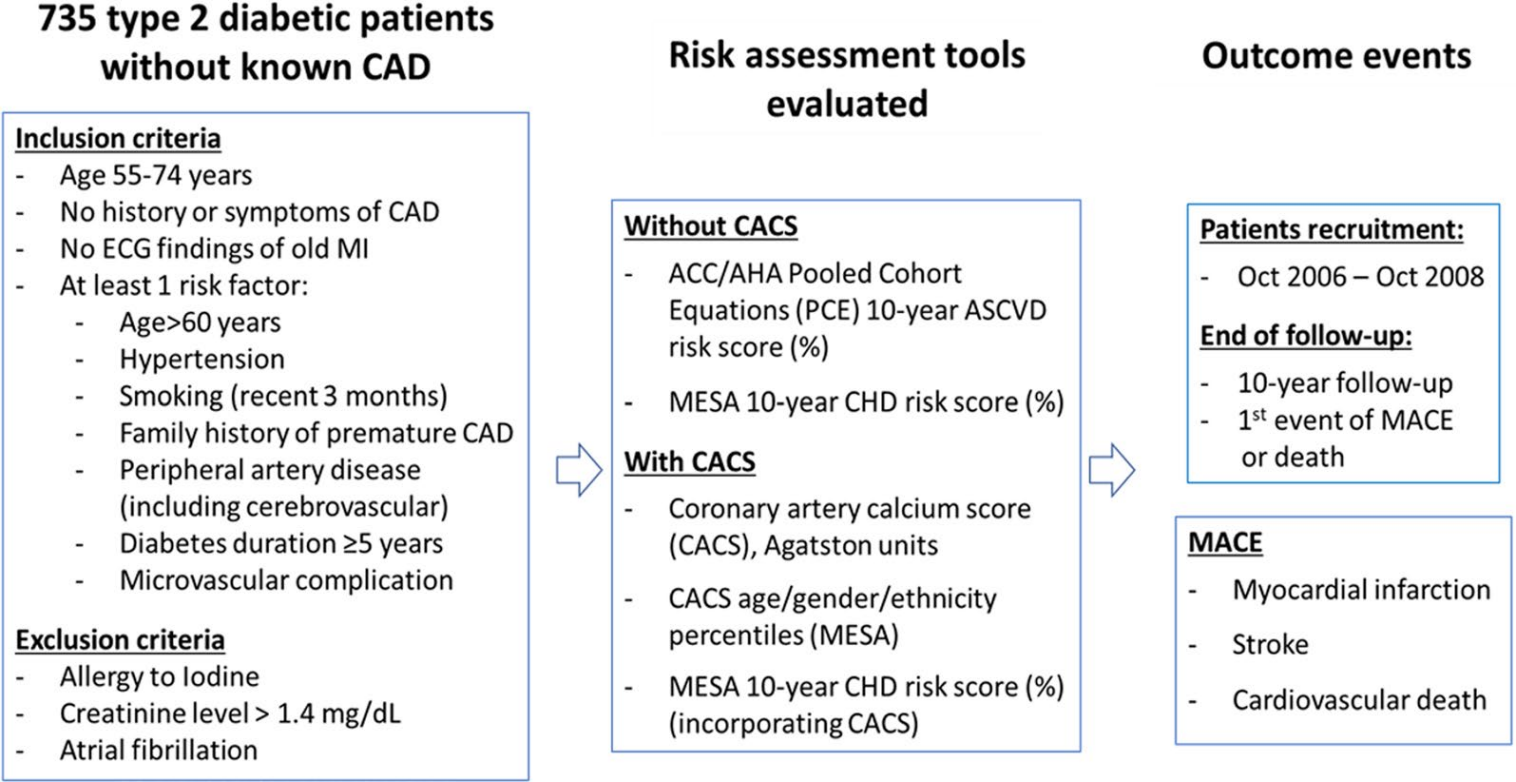
Study outline. CACS = coronary arteries calcium score; CAD = coronary artery disease; PCE = pooled cohort equations; MACE = major adverse clinical events; MESA = multi-ethnic study of atherosclerosis

This study is a retrospective analysis of a prospective study of cardiovascular outcomes in 735 asymptomatic type 2 diabetics enrolled between October 2006 and October 2008 who underwent CACS and cardiac CT angiography at study entry to examine coronary artery atheroma [10]. That study is registered at ClinicalTrials.gov as NCT00321542. Type 2 diabetics attending 2 regional diabetic or family practice clinics were screened for eligibility. Eligible patients providing informed consent were enrolled. Patients were included in the study if they fulfilled the following criteria: age 55–74 years, no history or symptoms of CAD or electrocardiographic signs of previous myocardial infarction (MI), and at least 1 cardiovascular risk factor, as elaborated in the study outline presented in Fig. 1. Exclusion criteria were serum creatinine > 1.4 mg/dL, allergy to iodine and chronic atrial fibrillation. All subjects were of Caucasian ethnicity. The primary outcome was major adverse cardiovascular events (MACE), defined as a composite of MI, stroke or cardiovascular death. Events were ascertained by phone calls and by reviewing patients' electronic files. End of follow-up was defined as first event of MACE or 10-year follow-up.

### Coronary artery calcification and risk scores

All study patients performed a baseline non-contrast enhanced chest CT scan for the measurement of CACS, performed using a 64-slice scanner (Brilliance-64, Philips Healthcare, Cleveland, Ohio). CACS was quantified using semi-automatic software (HeartBeat CS, Philips Medical Systems) that highlighted calcium deposits subsequently confirmed manually by the operator. Summed CACS was calculated by the system in Agatston units (AU) based on size and density of the calcium deposits [11].

Cardiovascular risk estimation was evaluated for each participant according to several risk assessment tools: (a) ACC/AHA PCE (based on age, sex, race, smoking status, systolic blood pressure, hypertension treatment status, diabetes status, and total and high-density lipoprotein cholesterol levels), estimating 10-year atherosclerotic cardiovascular disease (ASCVD) events (%), defined as first occurrence of nonfatal MI or coronary heart disease (CHD) death, or fatal or nonfatal stroke [7], (b) MESA 10-year CHD risk (based on age, sex, race/ethnicity, smoking status, diabetes status, family history of heart attack, systolic blood pressure, hypertension and lipid lowering treatment status, and total and high-density lipoprotein cholesterol levels), with or without CACS. MESA CHD events were defined as MI, resuscitated cardiac arrest, fatal CHD, and revascularization only if associated with adjudicated angina [8], (c) CACS percentiles, calculated according to reference values web tool, based on age, gender and ethnicity of participants in the MESA study [12]. CACS was additionally evaluated as a continuous variable [log (CACS + 1)] as well as 4 sequential CACS categories defined by traditional cut-off values. In order to examine the additional predictive value of CACS we examined the PCE and MESA risk scores as predictors of MACE with and without the addition of CACS.

The study protocol was approved by Carmel Medical Center (Haifa, Israel) institutional review board. Participants provided written informed consent.

### Statistical analysis

Continuous data are presented as means ± standard deviation or median and interquartile range (IQR), when appropriate, and categorical variables as numbers and percentages. Normality of distribution was assessed using the Kolmogorov–Smirnov test. Differences between normally distributed variables were evaluated using the independent-samples *T* test, and between non-normally distributed variables by the Mann–Whitney *U* test. The Chi-square test was used to compare categorical variables. Survival curves were plotted for each of the risk assessment tools by the Kaplan–Meier method using the log-rank test for comparison between subgroups. Univariable and multivariable Cox proportional hazards regression models were performed to evaluate the association of risk assessment tools and CACS with 10-year outcome events, with calculation of hazard ratios (HR) and 95% confidence intervals (CI). Adjustment was made for age, sex, duration of diabetes, insulin treatment, glycated hemoglobin, presence of retinopathy, nephropathy, neuropathy, creatinine clearance, prior stroke or transient ischemic attack, treatment with aspirin and statins, as well as clinical risk scores. For graphical presentation, a smoothed plot of adjusted HR (relative to CACS of zero) was estimated along with point-wise 95% CIs. For this purpose, the CACS was flexibly modeled in a Cox regression using restricted cubic spline function with five knots corresponding to approximately 5%, 25%, 50%, 75% and 95% percentiles of CACS [13]. Discriminatory capacity was assessed by calculating receiver operator characteristic (ROC) curves along with the area under the curve (AUC) for each of the risk assessment tools [14]. Binary logistic regression analysis was used to evaluate the predictive probability of the combination of risk markers. Improvement in discrimination was assessed by comparing AUCs of the different models using the method described by Delong et al. [15]. In addition, we examined the concordance between CACS and risk score models for predicting MACE through cross-tabulations of dichotomous categories. SPSS software version 25.0, MEDCALC version 19.2.1 and SAS version 9.4 were used to perform all statistical analyses.

## Results

### Patient characteristics

Study cohort included 735 type 2 diabetic patients aged 55–74 years with no history or symptoms of CAD. Mean age was 63 ± 5 years and 52% were women. Microvascular complications included retinopathy (17%), neuropathy (29%) and nephropathy (15%). The majority were treated with aspirin and statins at study entry (65% and 70%, respectively). Baseline clinical characteristics according to gender are presented in Table 1. CACS as well as PCE and MESA risk scores were higher in men than in women. CACS zero was observed in 15.5% of men and 31.2% of women.

**Table 1:** Baseline clinical characteristics

Variable	All (n = 735)	Men n = 354 (48.2%)	Women n = 381 (51.8%)	*P* value
Age (years)	63.4 ± 5.3	63.0 ± 5.2	63.8 ± 5.4	0.033
Diabetes, years since diagnosis	10.1 ± 7.6	10.2 ± 7.5	10.1 ± 7.7	0.794
Body Mass Index (BMI) (kg/m^2^)	29.4 ± 5.2	28.8 ± 4.2	30.0 ± 5.9	0.024
Insulin treatment	174 (23.7%)	70 (19.8%)	104 (27.3%)	0.017
Current smoking	105 (14.3%)	64 (18.1%)	41 (10.8%)	0.005
Hypertension	505 (68.7%)	232 (65.5%)	273 (71.7%)	0.074
Family history of CAD	182 (24.8%)	76 (21.5%)	106 (27.8%)	0.046
Prior CVA/TIA	48 (6.5%)	25 (7.1%)	23 (6.0%)	0.574
Retinopathy	124 (16.9%)	56 (15.8%)	68 (17.8%)	0.463
Neuropathy	216 (29.4%)	111 (31.4%)	105 (27.6%)	0.259
Nephropathy	108 (14.7%)	63 (17.8%)	45 (11.8%)	0.022
HbA1c (%)	7.47 ± 1.53	7.45 ± 1.54	7.50 ± 1.53	0.626
Hemoglobin (gm/dl)	13.5 ± 1.3	14.2 ± 1.2	12.8 ± 1.2	< 0.001
Creatinine	0.83 ± 0.18	0.93 ± 0.15	0.74 ± 0.14	< 0.001
Creatinine Clearance (per 1.73 sqM)	88.7 ± 20.1	86.4 ± 18.1	90.8 ± 21.6	0.012
Total cholesterol (mg/dl)	180.1 ± 36.1	174.8 ± 35.1	185.1 ± 36.3	< 0.001
HDL-C (mg/dl)	48.1 ± 12.0	43.5 ± 9.6	52.2 ± 12.5	< 0.001
Triglycerides (mg/dl)	172.4 ± 120.2	173.7 ± 135.5	171.1 ± 104.1	0.295
LDL-C (mg%)	98.6 ± 29.2	98.0 ± 28.4	99.1 ± 29.9	0.594
Aspirin	482 (65.6%)	249 (70.3%)	233 (61.2%)	0.009
Statins	519 (70.6%)	243 (68.6%)	276 (72.4%)	0.259
**Risk assessment tools**				
CACS, Agatston units	59 (1, 327)	164 (18, 501)	25 (0, 200)	< 0.001
0	174 (23.7%)	55 (15.5%)	119 (31.2%)	< 0.001
1–100	229 (31.2%)	99 (28%)	130 (34.1%)	
101–300	131 (17.8%)	65 (18.4%)	66 (17.3%)	
> 300	201 (27.3%)	135 (38.1%)	66 (17.3%)	
CACS Percentiles (MESA—age/gender /ethnicity adjusted)	70 (28, 86)	65.5 (41, 85)	72 (0, 88)	0.960
PCE 10-year Risk Score (%)	20.8 (12.4, 32.1)	27.1 (19.2, 37.1)	14.8 (8.7, 23.5)	< 0.001
PCE 10-year Risk Score > 20%	381 (51.8%)	259 (73.2%)	122 (32%)	< 0.001
MESA_(without CACS)_ 10-year risk score (%)	12.2 (8.3, 18.1)	17.6 (12.7, 23.1)	8.8 (6.4, 11.8)	< 0.001
MESA_(with CACS)_ 10-year risk score (%)	12.5 (5.2, 21.5)	19.1 (9.8, 26.7)	7.8 (3.7, 14.4)	< 0.001

CACS = coronary arteries calcium score; CAD = coronary artery disease; CVA = cerebrovascular accident; HbA1c = hemoglobin A1c; HDL-C = high-density lipoprotein cholesterol; LDL-C = low-density lipoprotein cholesterol; MESA = multi-ethnic study of atherosclerosis; PCE = pooled cohort equations; TIA = transient ischemic attack

Data presented as mean ± SD, median (IQR) or number (percent)

### Predictive power of risk assessment tools

**Fig. 2:**
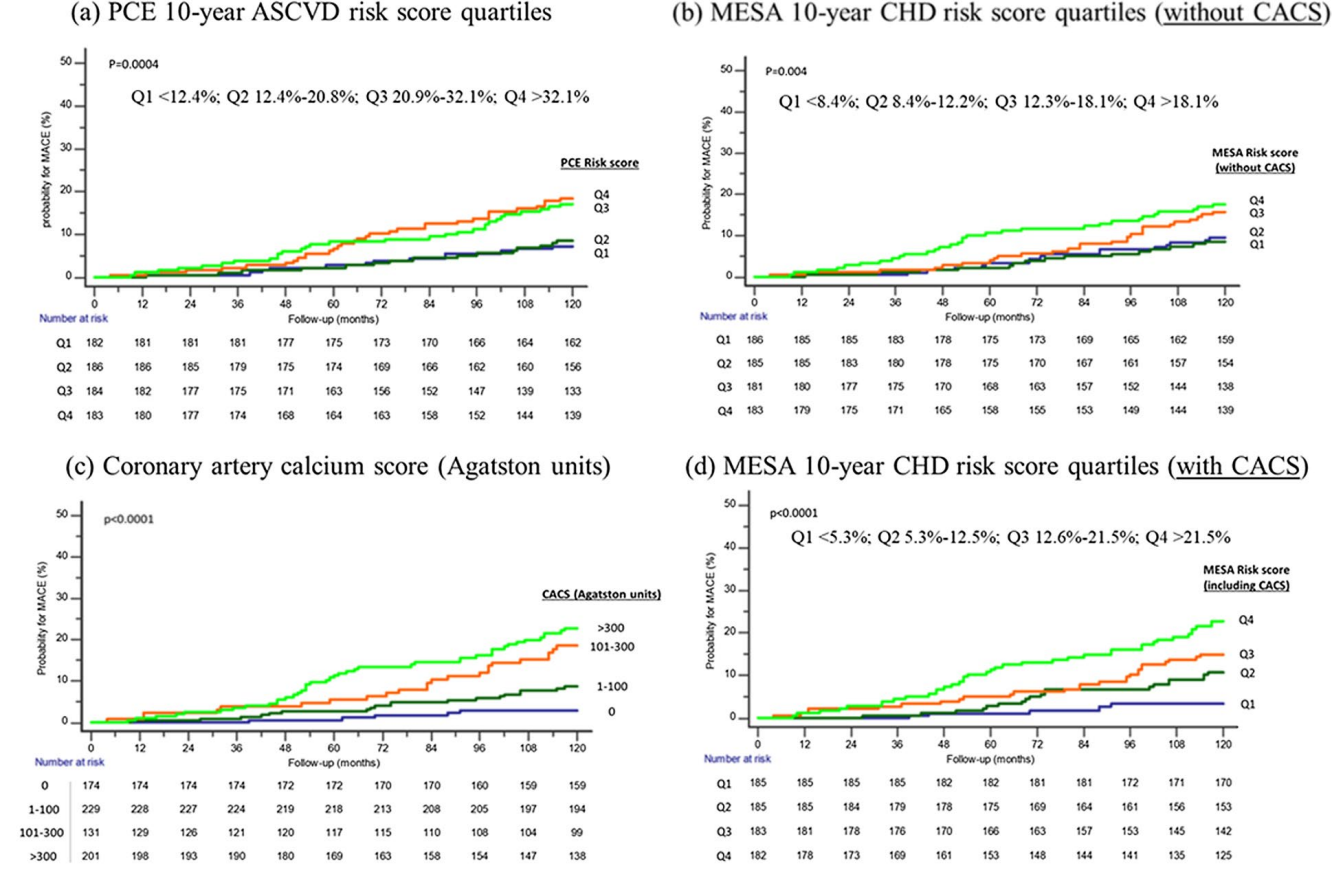
Kaplan–Meier curves presenting cumulative risk for 10-year MACE, according to the different risk assessment tools. CACS = coronary arteries calcium score; CHD = coronary heart disease; MACE = major adverse cardiovascular events; MESA = multi-ethnic study of atherosclerosis; PCE = pooled cohort equations

**Fig. 3:**
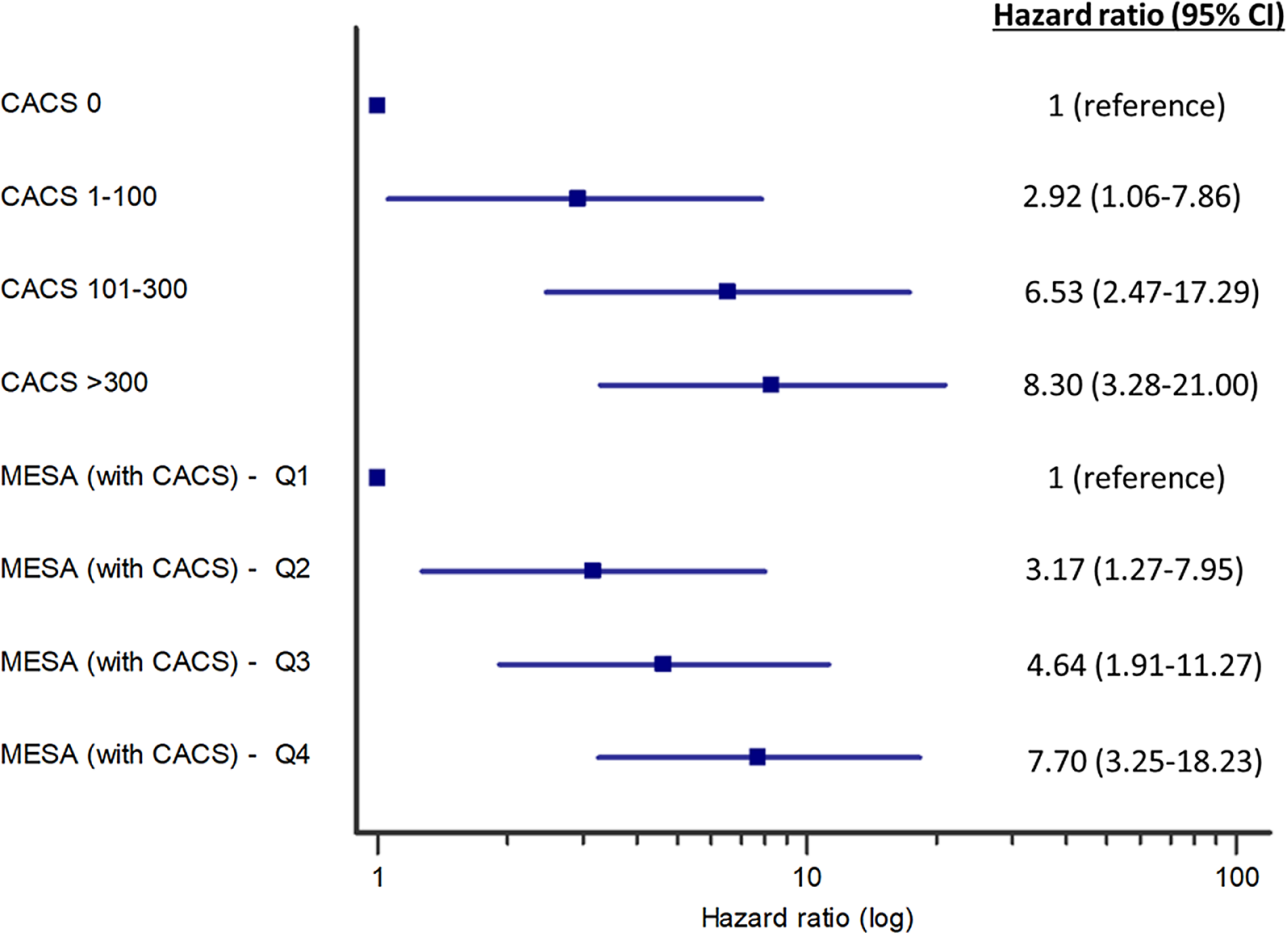
Adjusted hazard ratios for MACE associated with increase in coronary artery calcium scores. CACS = coronary arteries calcium score; CHD = coronary heart disease; CI = confidence interval; MACE = major adverse cardiovascular events; MESA = multi-ethnic study of atherosclerosis; PCE = pooled cohort equations; Q = quartiles. MESA 10-year CHD risk score incorporating CACS, presented by quartiles of percent risk. Hazard ratio was adjusted for duration of diabetes, insulin treatment, glycated hemoglobin, presence of retinopathy, nephropathy, neuropathy, creatinine clearance, prior CVA/TIA and medication treatment with aspirin or statin at enrolment. CACS, presented as a continuous variable (Agatston units). Hazard ratio was adjusted for previous variables as well as age, sex, PCE 10-year risk score (%) and MESA (without CACS) 10-year risk score (%)

**Fig. 4:**
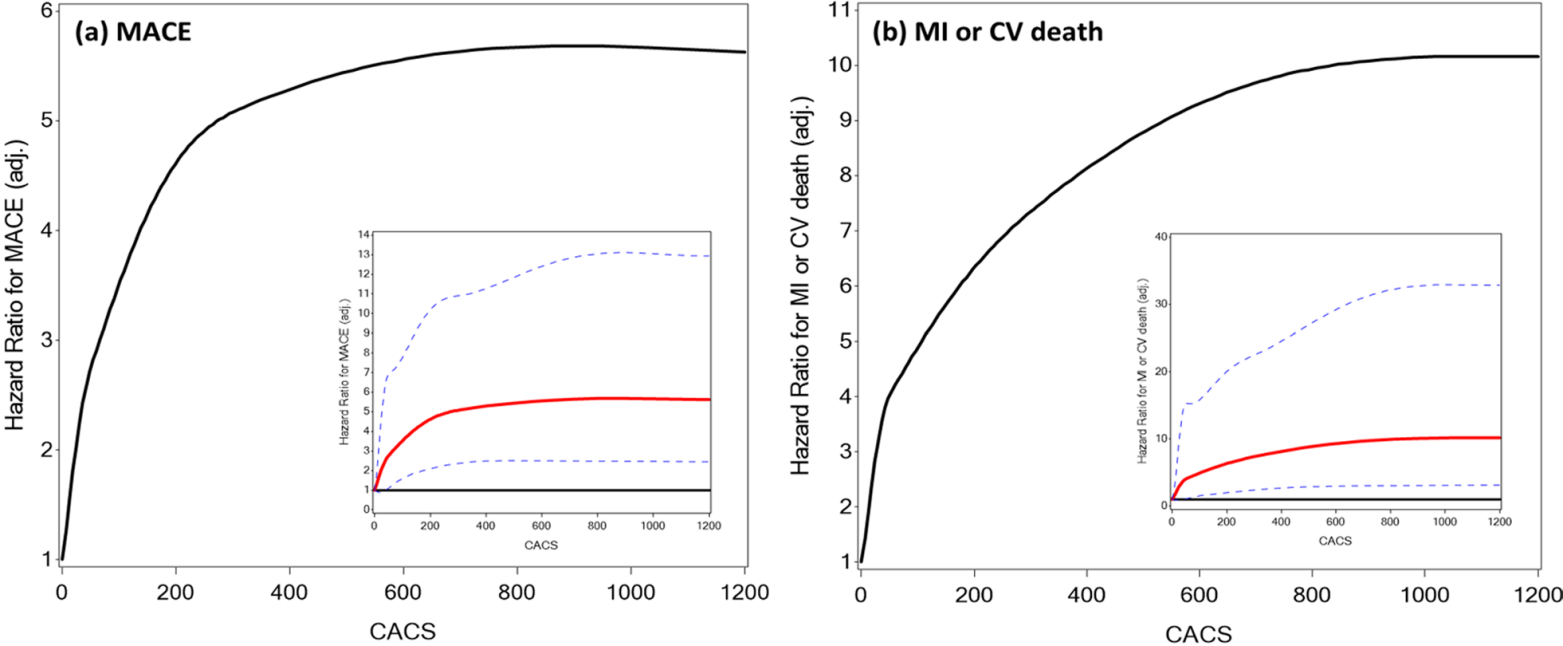
Adjusted hazard ratio for cardiovascular events associated with coronary artery calcium score, based on restricted spline model. For MACE outcome: test of any effect (linear + non-linear), *p* = 0.0003; test of the non-linear components, *p* = 0.0007; test of the linear component: *p*= 0.14. For MI/CV death outcome: test of any effect (linear + non-linear), *p* = 0.0011; test of the non-linear components, *p* = 0.0034; test of the linear component: *p* = 0.11. CACS = coronary arteries calcium score (Agatston units); CV = cardiovascular; MACE = major adverse cardiovascular events; MI = myocardial infarction

Measures of diabetes severity were associated with a significant increase in the risk for MACE, including the duration of diabetes, insulin therapy, glycated hemoglobin levels as well as microvascular complications (Additional file 1: Table S1). Similarly, the clinical risk assessment tools were associated with 10-year MACE: an increment of 1% in the PCE, MESA_(without CACS)_ or MESA_(with CACS)_ estimated risk scores were associated with a HR (95% CI) for MACE of 1.03 (1.01–1.04), 1.03 (1.01–1.05) and 1.04 (1.03–1.06), respectively, all *p* < 0.001 (Additional file 1: Table S2). CACS above the 75th percentile (according to age/gender/ethnicity MESA web tool) was associated with a HR of 2.63 95%CI (1.71–4.05), *p* < 0.001 for MACE compared to ≤ 75th percentile. Increase in CACS across sequential categories (0, 1–100, 101–300, > 300 AU) was also associated with significantly higher event rates of MACE as well as its individual components of MI, cardiovascular death, and to a lesser extent stroke (Table 2). Kaplan–Meier plots for MACE stratified by categories of the different risk scores and CACS show differential outcomes commencing after about 4 years of follow-up (Fig. 2 and Supplemental Figure S1). After multivariable adjustment, a gradual increase in the HR for MACE was observed across the quartiles of the MESA_(with CACS)_ risk score. In addition, CACS by itself was associated with a significant stepwise increase in the risk for MACE, after additional adjustment for clinical risk scores (Fig. 3). A smoothed restricted cubic spline plot further displays the effect of CACS on the adjusted HR for 10-year MACE (overall association *p* = 0.0003, non-linear association *p* = 0.0007), and MI or cardiovascular death (overall association *p* = 0.0011, non-linear association *p* = 0.0034). The HR rises rapidly with the initial increase in CACS, but more moderately with further increase in CACS (Fig. 4). In addition, a sensitivity analysis was performed based on presence or absence of several baseline predictors of MACE (Additional file 1: Table S3). The adjusted HR for MACE associated with a continuous increase in CACS was similar in the presence or absence of baseline treatment with statin, aspirin or insulin and with an age cutoff below or above the median (p-for-interaction non-significant for each comparison).

**Table 2:** Occurrence of cardiovascular events during 10-year follow-up, according to categories of coronary artery calcium score

CACS (Agatston units)		Event				
		MACE (n = 90)	MI (n = 36)	Stroke (n = 36)	CV death (n = 34)	MI or CV death (n = 62)
**0** (n = 174)	Proportion with event	5/174 (2.9%)	0	5/174 (2.9%)	0	0
	Rate per 1,000 person-years	2.96	0	2.96	0	0
	Hazard ratio*	1 (ref.)				
**1–100** (n = 229)	Proportion with event	19/229 (8.3%)	8/229 (3.5%)	6/229 (2.6%)	6/229 (2.6%)	13/229 (5.7%)
	Rate per 1,000 person-years	8.83	3.66	2.76	2.72	5.95
	Unadjusted hazard ratio	3.00 (1.12–8.04) *p* = 0.029				
	Adjusted hazard ratio	2.92 (1.06–7.86) *p* = 0.033				
**101–300** (n = 131)	Proportion with event	23/131 (17.6%)	6/131 (4.6%)	12/131 (9.2%)	9/131 (6.9%)	14/131 (10.7%)
	Rate per 1,000 person-years	19.74	4.94	10.07	7.23	11.53
	Unadjusted hazard ratio	6.78 (2.58–17.83) *p* < 0.001				
	Adjusted hazard ratio	6.53 (2.47–17.29) *p* < 0.001				
** > 300** (n = 201)	Proportion with event	43/201 (21.4%)	22/201 (10.9%)	13/201 (6.5%)	19/201 (9.5%)	35/201 (17.4%)
	Rate per 1,000 person-years	24.94	12.45	7.25	10.30	19.81
	Unadjusted hazard ratio	8.68 (3.44–21.90) *p* < 0.001				
	Adjusted hazard ratio	8.30 (3.28–21.00) *p* < 0.001				

CACS = coronary arteries calcium score (Agatston units); CV = cardiovascular; MACE = major adverse cardiovascular events; MI = myocardial infarction

^*^Hazard ratios are presented for MACE only due to wide confidence intervals secondary to low number of events in the individual endpoints. Adjustment was made for age, sex, duration of diabetes, insulin treatment, glycated hemoglobin, presence of retinopathy, nephropathy, neuropathy, creatinine clearance, prior CVA/TIA and medication treatment with aspirin or statin at enrolment, as well as PCE 10-year risk score (%) and MESA_(without CACS)_ 10-year risk score (%)

### The impact of CACS on risk reclassification

The HR for 10-year MACE and the event rates per 1,000 person years were significantly lower in patients with zero CACS, regardless whether the PCE 10-year ASCVD risk estimation was high (> 20%) or low/intermediate (< 20%) (Table 3). In addition, CACS > 75th percentile was associated with higher MACE rates in patients with both high (> 20%) or low/moderate (< 20%) PCE risk score. Discordance between MESA risk scores with compared to without CACS was also noted; MESA_(with CACS)_ levels < median were associated with reduced risk for MACE regardless whether MESA_(without CACS)_ risk estimation was below or above median levels. P-for-interactions were non-significant implying that the differential values of CACS (or MESA with CACS) in relation to MACE were of similar magnitude irrespective of the presence of a higher or lower PCE score or MESA_(without CACS)_.

**Table 3:** Incidence and hazard ratios for MACE, according to PCE and risk assessment tools including coronary artery calcium scoring

MACE	PCE < 20%		PCE > 20%		*p* value for-interaction
	CACS < 75%	CACS > 75%	CACS < 75%	CACS > 75%	
Proportion with event	10/207 (4.8%)	18/147 (12.2%)	22/215 (10.2%)	40/166 (24.1%)	
Rate pre 1,000 person-years	5.03	13.45	11.16	27.94	
Hazard ratio*	1 (reference)	2.71 (1.25–5.87) *p* = 0.011	2.24 (1.06–4.74) *p* = 0.034	5.71 (2.85–11.41) *p* < 0.001	0.813

MACE	PCE < 20%		PCE > 20%		
	CACS = 0	CACS > 0	CACS = 0	CACS > 0	
Proportion with event	3/119 (2.5%)	25/235 (10.6%)	2/55 (3.6%)	60/326 (18.4%)	
Rate pre 1,000 person-years	2.59	11.53	3.77	20.89	
Hazard ratio*	1 (reference)	4.51 (1.36–14.92) *p* = 0.014	1.46 (0.25–8.76) *p* = 0.676	8.25 (2.59–26.30) *p* < 0.001	0.894

MACE	MESA_(without CACS)_ < median		MESA_(without CACS)_ > median		
	MESA_(with CACS)_ < median	MESA_(with CACS)_ > median	MESA_(with CACS)_ < median	MESA_(with CACS)_ > median	
Proportion with event	21/280 (7.5%)	11/86 (12.8%)	3/87 (3.4%)	55/282 (19.5%)	
Rate pre 1,000 person-years	7.92	14.29	3.57	22.30	
Hazard ratio*	1 (reference)	1.83 (0.88–3.79) *p* = 0.105	0.45 (0.13–1.50) *p* = 0.193	2.87 (1.73–4.74) *p* < 0.001	0.074

*Unadjusted hazard ratio

CACS = coronary artery calcium score; MACE = major adverse cardiovascular events (myocardial infarction, stroke or cardiovascular death); MESA = multi-ethnic study of atherosclerosis; PCE = pooled cohort equations

### Discriminatory ability with and without CACS

**Fig. 5:**
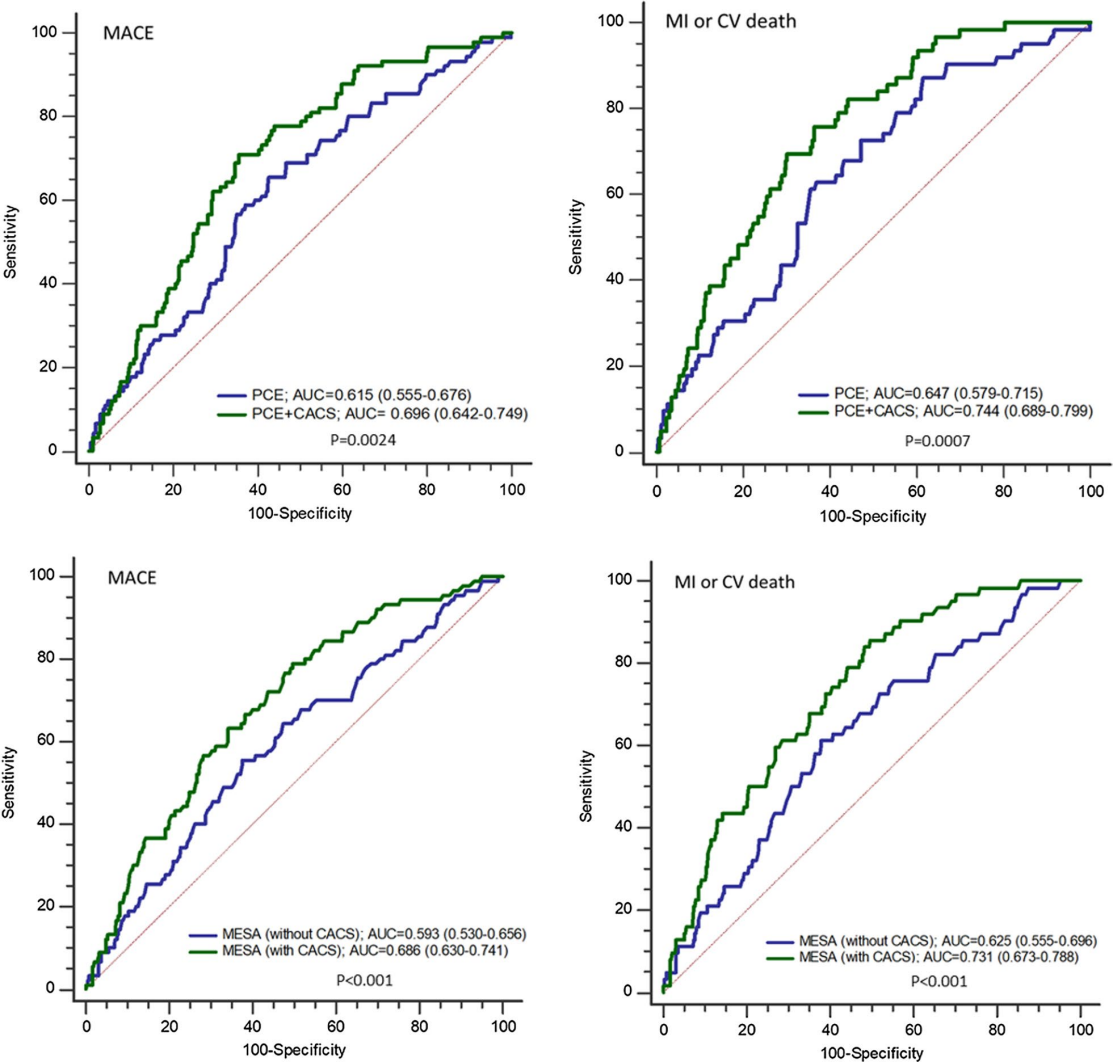
Additive discriminatory capacity with the addition of coronary artery calcium score. AUC = area under curve; CACS = coronary artery calcium score; CV = cardiovascular; PCE = pooled cohort equations; MESA = multi-ethnic study of atherosclerosis; MI = myocardial infarction. *p* Values represents the difference between pairs of curves

Additive discriminatory capacity for MACE was observed with the addition of CACS to both PCE risk score (AUC 0.696 vs. 0.615, *p* = 0.0024) and MESA risk score [MESA_(with CACS)_ AUC 0.686 vs. MESA_(without CACS)_ 0.593, *p* < 0.001) (Fig. 5). Similarly, the discriminatory capacity for MI or cardiovascular death significantly increased with the addition of CACS to PCE risk score (AUC 0.744 vs. 0.647, *p* = 0.0007) and MESA risk score [ (AUC 0.731 vs. 0.625, *p* < 0.001). Discriminatory ability was better for MI and cardiovascular death than for stroke (Additional file 1: Table S4).

## Discussion

In long-term follow-up of diabetic patients with additional risk factors but without known CAD, PCE and MESA risk scores predicted 10-year MI and cardiovascular death but not stroke. CACS was an independent predictor of MACE and each of its individual endpoints, improving discrimination of event risk prediction models. Absence of coronary artery calcification at study entry was evident in up to a quarter of the study population, and was associated with very-low event rates even in patients with high estimated clinical risk scores.

Diabetes mellitus was previously considered a CHD risk equivalent [16]. However, more recent data have shown that the CVD risk of patients with diabetes is markedly variable [17]. The present study population was comprised of middle-aged diabetic patients who did not have known CAD but had at least one additional cardiovascular risk factor. Half of the study cohort had an estimated PCE 10-year ASCVD risk of > 20%, considered a high-risk cut-off, as might be expected in diabetics. Notwithstanding, almost a quarter of the study population did not have any coronary artery calcification. CACS has significant prognostic value across a wide spectrum of risk factor profiles [4]. In particular, individuals who do not have any coronary artery calcification have low risk of cardiovascular events, and in this population CACS can serve as a “negative risk marker” [17, 18]. In the current study, among individuals classified at lower risk based on estimated PCE risk score < 20%, the presence of any CACS (> 0) was associated with four times higher event rates than in those with zero CACS. In contrast, among individuals traditionally identified as high risk based on estimated PCE risk score > 20%, CACS of zero was associated with remarkably low event rates. Of note, the PCE was previously reported to overestimate risk compared to the MESA multiethnic cohort of patients without baseline clinical CVD [19]. In the present analysis, the ability of low CACS to reclassify risk was further demonstrated by the MESA risk score estimation incorporating CACS. Malik and colleagues have analyzed diabetic patients participating in the MESA study, reporting that 38% of the patients with diabetes had a CACS of zero with annual CHD event rate similar to those without diabetes [20]. In a previous meta-analysis of 8 studies, CACS ≥ 10 AU in people with type 2 diabetes predicted all-cause mortality and cardiovascular events, suggesting that a finding of CACS < 10 may be clinically used to identify diabetic patients at low risk within this high-risk population [21]. Overall, these findings imply that CACS testing in diabetic patients could help reclassify risk of a significant proportion of patients to a lower risk group in the absence of coronary artery calcification.

Coronary artery calcification is often detected in asymptomatic individuals without known CVD. In a large cohort of asymptomatic individuals referred for CACS (only 7% diabetics), the addition of CACS to the PCE and to MESA_(without CACS)_ improved risk discrimination, particularly in the borderline and intermediate risk group [22]. Demonstration of subclinical atherosclerosis by CACS testing may benefit individuals who are at intermediate risk by traditional risk scores and have the greatest potential for risk reclassification and modification of outcome by intensification of preventive measures [23]. This may aid clinicians when evaluating the risk/benefit of preventive medications in diabetics, including the addition of aspirin which may be associated with adverse gastrointestinal effects, or the cost-effectiveness of PCSK9 monoclonal antibodies beyond statins [24]. In these cases, the use of CACS may improve the allocation of newer therapies and identify patients at higher risk who will have greater benefit in risk reduction. However, although diabetic patients with zero calcium have good long-term prognosis, and recent findings suggest that among individuals at intermediate risk with risk-enhancing factors, cardiovascular event rates are generally lower than the recommended threshold to initiate statin therapy when the CACS is zero [25], statins should be given to diabetic patients according to current clinical guidelines until prospective studies are performed. The presence and severity of CACS was repeatedly shown to improve discrimination and reclassification of future cardiovascular risk when compared to traditional risk factors, including in patients with type 2 diabetes [20, 26, 27]. Our findings further display the additive discriminatory capacity for MACE when adding CACS to clinical risk scores in diabetics. The discriminatory ability was even stronger when evaluating MI or cardiovascular death, excluding stroke as an outcome. CACS was shown in previous studies to serve as an independent predictor also for cerebrovascular events, improving discriminatory capacity [28, 29]. However, in a recent analysis of asymptomatic individuals, CACS was consistently a better predictor of CHD than stroke [30]. Of note, the MESA risk score was designed as predictor of CHD events and not stroke. Our findings are in line with these results, as both clinical risk calculators were predictors of stroke only if data on CACS was added, with a discriminatory ability that was far lower for stroke than that for MI or cardiovascular death.

Interestingly, in a restricted spline model, the adjusted HR for 10-year adverse events increased rapidly with the initial rise in CACS levels but became more moderate when CACS was > 400 AU for MACE and > 600 AU for MI or cardiovascular death. Although a wide confidence interval limits the interpretation of this analysis, it is possible that additional calcification of older plaques leads to their stabilization, while plaques with mild calcification are more active and prone to rupture or erosion, resulting in acute cardiovascular events [9, 31]. In this context, it should be noted that a significant proportion of the study population was treated with statins, which may have influenced the progression of CACS [32]. Statins were reported to increase coronary artery calcium density without increasing total calcium volume, which may stabilize existing plaque [33]. In addition, it was shown that CACS retains its predictive value among patients already on statin therapy, and therefore the utility of CACS testing should be maintained in diabetics, a population in whom the role of statins is unequivocal [34].

Strengths of this study include the prospective design and the recruitment of diabetic patients in a community-based setting under routine medical care. In addition, the availability of data on diabetes duration, baseline severity and complications, as well as long-term follow-up adds to the robustness of the results. Several limitations should be noted. First, patients with significant chronic kidney disease were excluded due to the use of CT angiography and the risk of contrast induced nephropathy. Moreover, most of our patients were under preventive medications at recruitment. These factors may impact on the generalizability of our findings. Second, the composite outcome event in the current study was not identical to that estimated by the PCE or MESA risk scores. Therefore, we did not investigate measures of calibration between models. Finally, the study was not designed to define treatment or to show benefit of routine screening on clinical outcomes. Treating physicians and patients received an assessment of risk as low, intermediate or high based on CACS. It is unknown whether this may have influenced clinician-patient behavior, and therefore impacted on clinical management.

## Conclusions

Although clinical risk scores may provide important prognostic information, CACS is an independent predictor of cardiovascular events that provides additive discriminatory capacity and an ability to reclassify risk of diabetic patients without known CAD. This enhances risk assessment and may help personalize the use of preventive medications and lifestyle interventions with a potential for improved outcomes.

## Supplementary Information

**Additional file 1**. Supplemental data.

## Data Availability

Not applicable.

## Abbreviations

AU: Agatston units
AUC: Area under the curve
CACS: Coronary artery calcium score
CAD: Coronary artery disease
CHD: Coronary heart disease
CI: Confidence interval
CT: Computed tomography
CVD: Cardiovascular disease
HR: Hazard ration
IQR: Interquartile range
MACE: Major adverse cardiovascular events
MESA: Multi-Ethnic Study of Atherosclerosis
MI: Myocardial infarction
PCE: Pooled Cohort Equations
ROC: Receiver operator characteristic curve
