# Supplementary material for: Value of addition of coronary artery calcium to risk scores in the prediction of major cardiovascular events in patients with type 2 diabetes
Source: BMC Cardiovasc Disord. 2021 Nov 13;21:541. doi: 10.1186/s12872-021-02352-4 (PMC8590310; doi:10.1186/s12872-021-02352-4)
Supplement: Supplementary file 1 — Additional file 1. Supplemental data. [file 12872_2021_2352_MOESM1_ESM.docx]

**Supplemental Data**

**Table S1**: Cox proportional hazards univariate association of patients’ characteristics with outcome events

| **Variable** | **MACE** | **MI** | **Stroke** | **CV death** |
| --- | --- | --- | --- | --- |
|  | HR (95% CI)  P value | HR (95% CI)  P value | HR (95% CI)  P value | HR (95% CI)  P value |
| Age (years) | **1.06 (1.02-1.10)**  **P=0.003** | 1.03 (0.97-1.09)  P=0.348 | 1.02 (0.96-1.09)  P=0.513 | **1.15 (1.08-1.23)**  **P<0.001** |
| Sex (Male) | 1.14 (0.76-1.73)  P=0.529 | 1.93 (0.98-3.81)  P=0.058 | 1.09 (0.57-2.09)  P=0.800 | 0.85 (0.43-1.68)  P=0.645 |
| Obesity (BMI >30 kg/m^2^) | 0.98 (0.64-1.49)  0.922 | 0.73 (0.37-1.47)  P=0.380 | 1.18 (0.61-2.28)  P=0.621 | 1.31 (0.67-2.57)  P=0.430 |
| DM, years since diagnosis (per 1yr) | **1.05 (1.03-1.07)**  **P<0.001** | **1.04 (1.01-1.08)**  **P=0.025** | **1.04 (1.00-1.08)**  **P=0.050** | **1.07 (1.03-1.11)**  **P<0.001** |
| DM, years since diagnosis  (<10 yrs vs. ≥10 yrs) | **1.97 (1.29-3.02)**  **P=0.002** | 1.50 (0.78-2.89)  P=0.228 | 1.65 (0.85-3.21)  P=0.137 | **3.31 (1.54-7.09)**  **P=0.002** |
| Insulin treatment | **2.06 (1.34-3.15)**  **P=0.001** | 1.47 (0.72-2.98)  P=0.291 | **2.69 (1.39-5.19)**  **P=0.003** | **2.34 (1.18-4.63)**  **P=0.015** |
| HbA1c (%) | **1.19 (1.05-1.33)**  **P=0.004** | 1.18 (0.98-1.43)  P=0.073 | 1.12 (0.92-1.36)  P=0.245 | **1.30 (1.08-1.55)**  **P=0.004** |
| Current smoking | 1.51 (0.89-2.55)  P=0.129 | 1.81 (0.83-3.98)  P=0.138 | - 1. (0.39-2.60)   P=0.982 | 0.84 (0.30-2.39)  P=0.744 |
| Hypertension | 1.21 (0.76-1.92)  P=0.414 | 1.05 (0.52-2.14)  P=0.891 | 1.40 (0.66-2.98)  P=0.382 | 1.51 (0.69-3.34)  P=0.306 |
| Family history of CAD | 1.44 (0.93-2.24)  P=0.106 | **2.40 (1.24-4.63)**  **P=0.009** | 0.72 (0.31-1.63)  P=0.427 | 1.06 (0.49-2.26)  P=0.887 |
| Prior CVA/TIA | **3.28 (1.89-5.71)**  **P<0.001** | 2.47 (0.96-6.36)  P=0.061 | **3.84 (1.68-8.76)**  **P=0.001** | **3.91 (1.70-8.97)**  **P=0.001** |
| Retinopathy | **2.04 (1.30-3.22)**  **P=0.002** | 1.43 (0.65-3.14)  P=0.373 | 1.67 (0.79-3.55)  P=0.182 | **3.52 (1.78-6.97)**  **P<0.001** |
| Neuropathy | **1.88 (1.24-2.85)**  **P=0.003** | **1.94 (1.01-3.75)**  **P=0.048** | **1.95 (1.01-3.75)**  **P=0.047** | **2.15 (1.09-4.21)**  **P=0.026** |
| Nephropathy | **1.84 (1.13-3.00)**  **P=0.014** | 1.46 (0.64-3.33)  P=0.372 | 1.72 (0.78-3.77)  P=0.177 | **3.36 (1.66-6.79)**  **P=0.001** |
| Total cholesterol (per 10 mg/dL increase) | 0.99 (0.94-1.05)  P=0.768 | - 1. (0.96-1.14)   P=0.364 | 0.99 (0.90-1.08)  P=0.767 | 0.97 (0.88-1.07)  P=0.501 |
| HDL-C (per 5 mg/dL reduction) | 1.04 (0.95-1.14)  P=0.439 | 1.13 (0.96-1.32)  P=0.132 | 0.98 (0.86-1.12)  P=0.778 | - 1. (0.91-1.24)   P=0.437 |
| Cholesterol/HDL-C Ratio | 1.04 (0.88-1.22)  P=0.663 | 1.21 (0.99-1.48)  P=0.062 | 0.96 (0.72-1.28)  P=0.771 | - 1. (0.78-1.34)   P=0.889 |
| Non-HDL Cholesterol (pre 10 mg/dL increase) | 1.00 (0.94-1.06)  P=0.972 | 1.06 (0.98-1.16)  P=0.155 | 0.98 (0.89-1.08)  P=0.693 | 0.98 (0.89-1.08)  P=0.678 |
| Aspirin therapy | 1.47 (0.92-2.34)  P=0.109 | 1.87 (0.85-4.09)  P=0.120 | 1.20 (0.59-2.43)  P=0.621 | **2.46 (1.02-5.95)**  **P=0.045** |
| Statin therapy | 1.22 (0.76-1.97)  P=0.403 | 1.25 (0.59-2.66)  P=0.562 | 1.73 (0.76-3.96)  P=0.191 | 1.34 (0.61-2.96)  P=0.467 |

CI, confidence interval; HR, hazard ratio; Q, quartile

**Table S2**: Cox proportional hazards univariate association of risk assessment tools with outcome events

| **Variable** | **MACE** | **MI** | **Stroke** | **CV death** |
| --- | --- | --- | --- | --- |
|  | HR (95% CI)  P value | HR (95% CI)  P value | HR (95% CI)  P value | HR (95% CI)  P value |
| PCE 10-year risk score (per 1% increase) | **1.03 (1.01-1.04)**  **P<0.001** | **1.03 (1.01-1.05)**  **P<0.001** | - 1. (0.99-1.04)   P=0.343 | **1.04 (1.02-1.06)**  **P<0.001** |
| PCE 10-year risk score (median) | **2.39 (1.53-3.73)**  **P<0.001** | **2.75 (1.33-5.70)**  **P=0.007** | 1.87 (0.95-3.70)  P=0.071 | **3.44 (1.56-7.59)**  **P=0.002** |
| MESA_(without CACS)_ 10-year risk score (per 1% increase) | **1.03 (1.01-1.05)**  **P<0.001** | **1.04 (1.02-1.07)**  **P<0.001** | 1.00 (0.97-1.04)  P=0.816 | - 1. (0.996-1.06)   P=0.092 |
| MESA_(without CACS)_ 10-year risk score (median) | **1.89 (1.23-2.91)**  **P=0.004** | **3.62 (1.65-7.94)**  **P=0.001** | 1.28 (0.67-2.48)  P=0.457 | 1.88 (0.93-3.80)  P=0.079 |
| MESA_(with CACS)_ 10-year risk score (per 1% increase) | **1.04 (1.03-1.06)**  **P<0.001** | **1.06 (1.04-1.08)**  **P<0.001** | 1.02 (0.99-1.05)  P=0.124 | - 1. **(1.02-1.06)**   **P<0.001** |
| MESA_(with CACS)_ 10-year risk score (median) | **3.02 (1.89-4.82)**  **P<0.001** | **4.48 (1.96-10.22)**  **P<0.001** | 1.89 (0.96-3.73)  P=0.067 | **4.96 (2.05-11.97)**  **P<0.001** |
| Log_10_(CACS+1) | **1.95 (1.54-2.46(**  **P<0.001** | **2.72 (1.75-4.22)**  **P<0.001** | **1.51 (1.09-2.10)**  **P=0.014** | **2.39 (1.56-3.66)**  **P<0.001** |
| CACS age/gender/ethnicity percentiles (MESA) (>75% vs. ≤75%) | **2.63 (1.71-4.05)**  **P<0.001** | **3.70 (1.78-7.67)**  **P<0.001** | **2.22 (1.13-4.33)**  **P=0.020** | **2.92 (1.42-5.98)**  **P=0.004** |

CI, confidence interval; HR, hazard ratio; Q, quartile

**Table S3**: Multivariable adjusted hazard ratios for MACE, associated with coronary artery calcium score*, in different subgroup populations

| **Variable** | **Proportion with event** | **MACE**    **HR (95% CI)**  **P value** | **P for interaction** |
| --- | --- | --- | --- |
| Overall population | 90/735 (12.2%) | 1.95 (1.53-2.47)  P<0.001 |  |
| Statin - no | 23/214 (10.7%) | 1.61 (1.07-2.41)  P=0.022 | 0.528 |
| Statin - yes | 67/519  (12.9%) | 2.07 (1.52-2.80)  P<0.001 |  |
| Aspirin - no | 24/253  (9.5%) | 1.50 (1.01-2.22)  P=0.045 | 0.135 |
| Aspirin - yes | 66/482  (13.7%) | 2.23 (1.64-3.04)  P<0.001 |  |
| Insulin - no | 56/561  (10%) | 1.83 (1.34-2.49)  P<0.001 | 0.729 |
| Insulin - yes | 34/174  (19.5%) | 1.78 (1.21-2.62)  P=0.004 |  |
| Age (years) <median | 39/367  (10.6%) | 1.62 (1.17-2.24)  P=0.004 | 0.439 |
| Age (years) >median | 51/368  (13.9%) | 2.27 (1.52-3.39)  P<0.001 |  |

CACS, coronary artery calcium scoring; CI, confidence interval; HR, hazard ratio

* CACS (Agatston units) was evaluated as a continuous variable: [log (CACS+1)]

Adjustment was performed for age, sex, duration of diabetes, insulin treatment, glycated hemoglobin, presence, of retinopathy, nephropathy, neuropathy, creatinine clearance, prior CVA/TIA, PCE 10-year risk score (%), MESA (without CACS) 10-year risk score (%), and medication treatment with aspirin or statin at enrolment (excluding the main variable tested in each analysis).

**Table S4:** Comparison of discriminatory capacity of event risk prediction models

| **Variable** | **MACE** | **MI** | **Stroke** | **CV death** | **MI or CV death** |
| --- | --- | --- | --- | --- | --- |
|  | AUC (95% CI) | AUC (95% CI) | AUC (95% CI) | AUC (95% CI) | AUC (95% CI) |
| **HbA1C (%)** | 0.593 (0.530-0.655)  P=0.004 | 0.584 (0.483-0.684)  P=0.090 | 0.560 (0.465-0.655)  P=0.223 | 0.645 (0.547-0.742)  P=0.004 | 0.589 (0.512-0.666)  P=0.020 |
| **Diabetes duration (years)** | 0.612 (0.546-0.679)  P=0.001 | 0.577 (0.479-0.674)  P=0.120 | 0.577 (0.465-0.688)  P=0.121 | 0.679 (0.587-0.771)  P<0.001 | 0.633 (0.557-0.708)  P=0.001 |
| **PCE** | 0.615 (0.555-0.676)  P<0.001 | 0.623 (0.536-0.711)  P=0.012 | 0.554 (0.463-0.646)  P=0.270 | 0.667 (0.581-0.754)  P=0.001 | 0.647 (0.579-0.715)  p<0.001 |
| **PCE + CACS** | 0.696 (0.642-0.749)  P<0.001 | 0.741 (0.673-0.810)  P<0.001 | 0.611 (0.524-0.697)  P=0.023 | 0.738 (0.665-0.812)  P<0.001 | 0.744 (0.689-0.799)  P<0.001 |
| Change in AUC*  P value | 0.081  P=0.0024 | 0.118  P=0.0008 | 0.056  P=0.306 | 0.071  P=0.045 | 0.097  P=0.0007 |
| **MESA_(without CACS)_** | 0.593 (0.530-0.656)  P=0.032 | 0.663 (0.577-0.749)  P=0.001 | 0.523 (0.427-0.619)  P=0.049 | 0.598 (0.509-0.687)  P=0.054 | 0.625 (0.555-0.696)  P=0.001 |
| **MESA_(with CACS)_**  Change in AUC*  P value | 0.686 (0.630-0.741)  P<0.001 | 0.762 (0.691-0.833)  P<0.001 | 0.596 (0.508-0.685)  P=0.051 | 0.699 (0.625-0.773)  P<0.001 | 0.731 (0.673-0.788)  P<0.001 |
|  | 0.093  P<0.001 | 0.099  P<0.001 | 0.073  P=0.021 | 0.101  P=0.0004 | 0.106  P<0.001 |
| **CACS**  **(Agatston units)** | 0.687 (0.635-0.740)  P<0.001 | 0.738 (0.672-0.804)  P<0.001 | 0.608 (0.521-0.695)  P=0.028 | 0.724 (0.652-0.796)  P<0.001 | 0.735 (0.681-0.789)  P<0.001 |

AUC, area under the curve; CACS, coronary arteries calcium score [score calculated as log(CAC+1)]; CI, confidence interval; CV, cardiovascular; MACE, major adverse cardiovascular events (myocardial infarction, stroke or cardiovascular death); MESA, multi-ethnic study of atherosclerosis; MI, myocardial infarction; PCE, pooled cohort equations

* Change in AUC was calculated by Delong et al. (ref. 19).

**Figure S1:** Kaplan-Meier curves presenting cumulative risk for 10-year MACE, according to percentiles of coronary artery calcium scores*

**
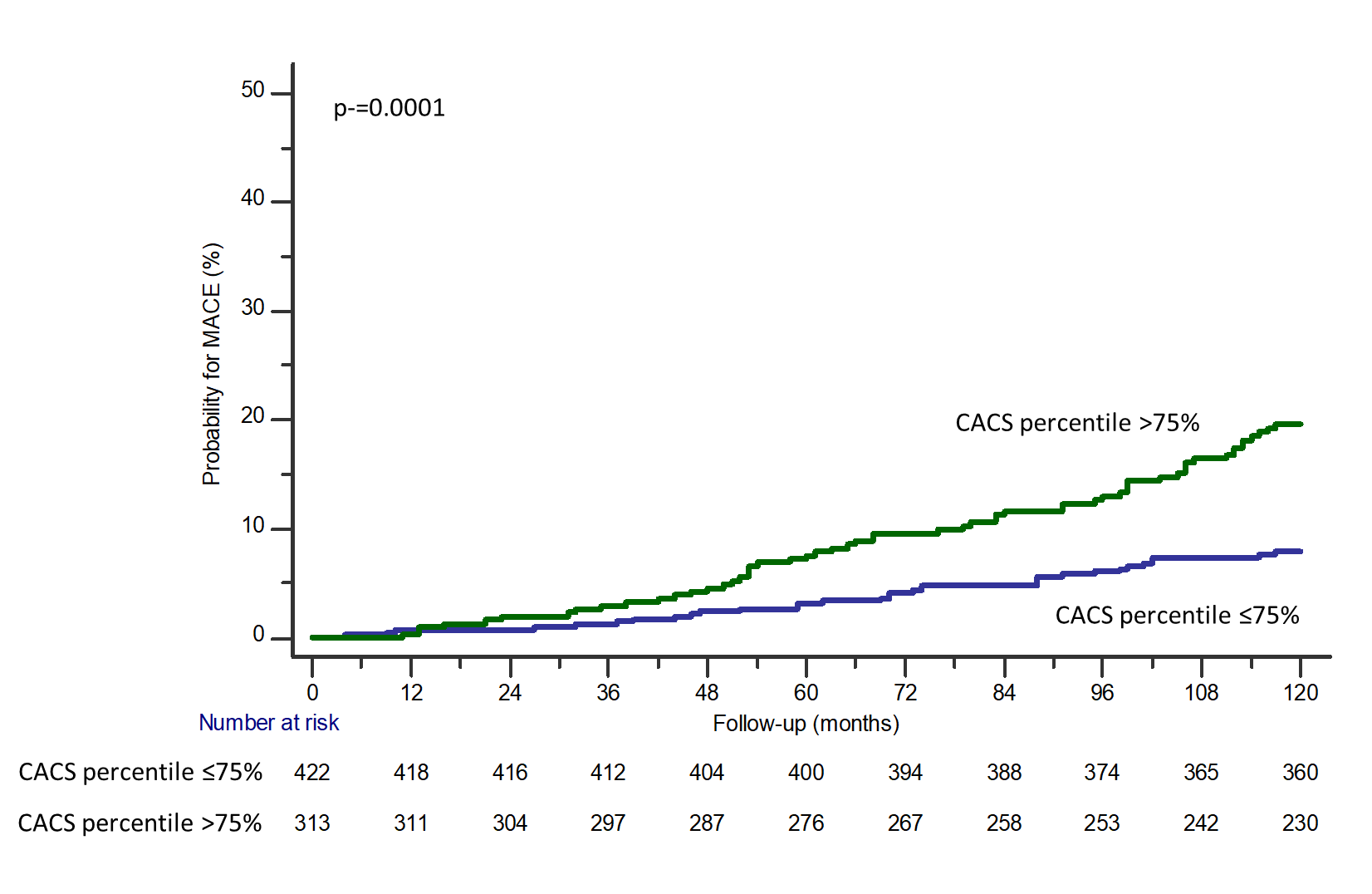
**

* Age, gender and ethnicity adjusted
